# Food insecurity is an important risk factor for type 2 diabetes: a case-control study of new referrals to the University clinics, Shiraz, Southern Iran

**DOI:** 10.1186/s12889-019-7236-9

**Published:** 2019-07-05

**Authors:** Narjes Najibi, Roya Firoozi, Shaghayegh Shahrezaee, Mohammadreza Eshraghian, Milad Daneshi-Maskooni, Ahmadreza Dorosty-Motlagh

**Affiliations:** 1 Department of Community Nutrition, School of Nutritional Sciences and Dietetics, Tehran University of Medical Sciences, Tehran, Iran; 2 Department of Nutritional Sciences, Varastegan Institute for Medical Sciences, Mashhad, Razavi Khorasan Iran; 3 Department of Biostatistics and Epidemiology, School of Public Health, Tehran University of Medical Sciences, Tehran, Iran; 4 School of Medicine, Jiroft University of Medical Sciences, Jiroft, Kerman Iran

**Keywords:** Food insecurity, Socioeconomic factors, Type 2 diabetes, Iran

## Abstract

**Background:**

The prevalence of food insecurity (FI) as “the limited or uncertain availability of enough food for an always active and healthy life” and diabetes as “the most common metabolic disease” are rising in Iran. The aim was to assess the FI, depression, and socioeconomic status as risk factors for type 2 diabetes (T2D).

**Methods:**

This case-control study was conducted on 135 patients with T2D as cases (99 females, 36 males, mean age 46.83 years) and 135 subjects without diabetes (89 females, 46 males, mean age 45.93 years) as controls. They had been referred to clinics of Shiraz University of Medical Sciences, Shiraz, Iran. The prior major inclusion criterion for diabetes was fasting blood sugar (FBS) ≥126 mg/dl. General, demographic, and socioeconomic characteristics and FI status were assessed using the general and 18-items United States Department of Agriculture (USDA) household food security questionnaires, respectively. Chi-square, t-test, and uni-and multi-variate logistic regression tests and SPSS_16_ statistical software were used.

**Results:**

The prevalence of FI was 66.7% in cases and 41.5% in controls. According to final analysis model, FI (Odds Ratio [OR] = 1.9, *P* = 0.016), depression (OR = 2.0, *P* = 0.018), body mass index (BMI) ≥ 25 kg/m^2^ (OR = 1.8, *P* = 0.025), number of children ≥4 (OR = 1.7, *P* = 0.046), and having children under 18 years. (OR = 2.1, *P* = 0.011) were significant independent risk factors for T2D.

**Conclusion:**

The prevalence of FI in patients with T2D was significantly higher compared to the controls. FI was an important risk factor for T2D, even after controlling for the potential confounders. Further studies are suggested.

## Background

Food insecurity (FI) as the lack of enough food for an active and healthy life [1–3] ranges from concern about availability and accessibility of food at the household level to starvation among children. Although FI and famine occur from financial constraints, the measurement of poverty and income data would not give clear information on the household food security status [4]. Food and nutrition are fundamental needs in the mental and physical health of humans [5]. FI has the different potential health consequences including overweight or obesity, chronic diseases, hypertension, dyslipidemia, mental disorders especially depression [3, 6], cancer [2, 7, 8], and low intelligence quotient [9]. The Economic Research Service (ERS) of USDA reported about half of FI is in Asia and likely near to 20% of them are faced with hunger [2]. According to previous studies, FI is affected by various factors such as the age, status of the household head, economic level, the employment status, the ethnicity, the family size, and the dietary habits [10].

T2D as one of the most common metabolic diseases and a World Health Organization (WHO) priority is related to different damages, chronic diseases [11, 12], and dietary fats [13, 14]. It estimated the prevalence of T2D in Iran about 7% up to 2025 [15]. However, the recent prevalence of diabetes in Iranian adults was 8.9% [16].

Budget limitations in food insecure individuals lead to buying cheaper and high dense calorie foods; which might contribute to obesity, and increased susceptibility to chronic illnesses including depression, T2D, and other medical condition [3]. Two previous studies in Canada and the USA have described the relationship between FI and diabetes [10, 17].

According to recent studies, the prevalence of FI [17–25] and the T2D [15, 26, 27] are rising in Iran. Due to the adverse consequences of FI and diabetes on health and no previous Iranian studies on FI as a risk factor for T2D, this study was designed to assess FI and associated socioeconomic factors and depression as risk factors for T2D among the newly diagnosed patients.

## Methods

This case-control study was conducted on 135 patients (99 women and 36 men, average age of 46.83 years) with T2D as cases and 135 similar participants without T2D (89 women and 46 men, average age of 45.93 years) as controls in 2012. The locations of random sampling were clinics of Shiraz University of Medical Sciences, Shiraz, Iran.

In the beginning, a pilot study was done on 40 newly diagnosed patients with T2D to calculate the sample size, know the conditions, and determine the accuracy. According to the pilot study in four clinics called *Ghamar-e-Banihashem*, *Nader Kazemi*, *Yaghtin*, and *Hazrat-e-Abolfazl*, the prevalence of FI in the participants with and without T2D was 60% (P_1_) and 42% (P_2_), respectively. The type I and type II errors were considered 0.05 and 0.2. The sample size was calculated 122 subjects by the proper formula. Also, the sample size was calculated 29 subjects according to the formula and the prevalence of depression in previous studies as 11.3 and 40.6% in participants with and without T2D [28]. Thus, it was determined a 135 subject sample for each group by considering an 8% dropout rate (the lack of cooperation until the end of the questioning). However, the dropout rate was zero at the end.

From 12 diabetes screening clinics, four above-mentioned clinics were randomly selected. The inclusion criteria were the age 30–60 years, fasting blood sugar (FBS) ≥ 126 mg/dl and newly diagnosed T2D (within less than 1 month) for the cases, and FBS < 126 mg/dl for the controls. The exclusion criteria were a history of the cardiovascular diseases, cancer, diabetes within more than0 1 month, and any medication for hypertension, dyslipidemia, and diabetes, any dietary changes, pregnancy or breastfeeding, and not informed consent for the enrollment.

First, the patients with T2D according to the screening, lab results, and eligibility criteria were identified and referred to the principal investigator as the interviewer. The clinics were located in different parts of Shiraz and included different social and economic classes. After necessary explanations about the study and signing the informed consent form, the questionnaires including the general, food security status, Beck depression, and MET physical activity were completed by interviewing, respectively. The general questionnaire included the demographic and socioeconomic characteristics such as age, marital status, employment status, education level, number of children, family size, having children under 18-year-old, monthly income, menopause status, and the type of oil consumed. The food security status was determined using the 18-item USDA household food security questionnaire [29] that is valid and reliable in Iran [18, 22]. The scoring method for this questionnaire was: a positive score (score 1) to answers “often”, “sometimes”, “almost every month”, “some months”, and “yes” and score 0 to answers “not correct”, “refused or didn’t know”, “only once or twice a month”, and “no” (Table 1) [29]. This study lasted from 24 September 2012 until 22 August 2012. The duration of the study was nearly 6 months.

**Table 1:** The household food security status based on the food security questionnaire final score

Food security status	code	Number of positive answers	
		Families with children under 18 (total score: 18)	Families without children under 18 (total score: 10)
Food secure	0	0–2	0–2
Food insecure without hunger	1	3–7	3–5
Food insecure with moderate hunger	2	8–12	6–8
Food insecure with severe hunger	3	13–18	9–10

Guide to Measuring Household Food Security (Bickel et al, 2000) [29]

The Beck depression questionnaire for assessing depression status was the usual form with 21 items. Beck is a self-report test to measure the intensity of depression. The questions are scored from 0 to 3 and the overall score is determined by summing all of the scores and ranged between 0 and 63. According to Beck, scores 0–9 is considered as no depression, 10–18 mild depression, 19–29 moderate depression, and 30–63 severe depression (Table 2). In this study according to Beck et al. [30] scores ≥19 were considered as depressed. This questionnaire is valid and reliable around the world (Cronbach’s alpha between 0.73–0.92 [30, 31] and 0.92 in Iran [32]), doesn’t need the specialized knowledge for carrying out, and can be completed in a short time.

**Table 2:** The classification of depression status based on scores of the Beck questionnaire

Depression status	Total score
No depression	0–9
Mild depression	10–18
Moderate depression	19–29
Severe depression	30–63

The weight was measured using the *Germany Seca-760*® analog scale with a 100-g accuracy with minimum coverage and without shoes, the height was measured using the tape measure on the wall with a 0.5 cm accuracy according to standard instructions. The body mass index (BMI) was calculated using dividing the weight (kg) into the square of height (meter) (as weight/height^2^). The BMI of subjects with normal weight and overweight/obesity was between 18.5–24.9 kg/m2 and ≥ 25 kg/m2, respectively.

The classified physical activity questionnaire was based on the metabolic equivalents (METs) of tasks with 9 activity levels from rest and sleep (METs = 0.9) to severe activity (METs> 6) [33]. Activities with MET< 3 were considered as sedentary activities, with MET between 3 and 6 as moderate activities, and with MET> 6 as severe activities. This questionnaire was valid and reliable in Iran [34]. The design of it is simple with 9 rows according to the intensity of physical activity from up to down as METs 0.9, 1, 1.5, 2, 3, 4, 5, 6, and > 6. The amount of activity as MET.Hour/day and the weight were used to determine the expended energy of activities per day.

Given that the type of dietary oil affects the incidence of metabolic disorders including diabetes [14], it was assessed as a risk factor.

The chi-square, t-test, and univariate logistic regression tests and the statistical software SPSS_16_ were used. Finally, all of the significant statistically factors in the univariate analysis models were entered backward to the multivariate logistic regression model to control the likely confounders and identify the important independent risk factors. The *p*-value ≤0.05 was statistically considered significant.

## Ethical considerations

This case-control study was approved by the ethics committee of the Tehran University of Medical Sciences (Registration No. 146).

## Results

The gender distribution was 73.3% female and 26.7% male in the patients with T2D and 65.9% female and 34.1% male in the participants without T2D. The average age was 46.83 years in cases and 45.93 years in controls. The age and gender between the two groups were nearly similar, although matching has not been done for participants at the beginning.

The marital and menopause status, age, gender, height, and employment status were not significantly related to T2D in newly diagnosed patients (Table 3).

**Table 3:** General characteristics, socioeconomic factors, anthropometric measurements, consumed oil type, and physical activity status in the participants with and without type 2 diabetes (T2D)

Variables			Cases	Controls	OR (95% CI)	*P*-value
			Number (%) or Mean ± SD	Number (%) or Mean ± SD		
Food security status	Food secure		45 (33.3)	79 (58.5)	1	< 0.001* < 0.001^○^
	Food insecure	Food insecure without hunger	64 (47.5)	51 (37.8)	2.8 (1.71–4.63)	
		Food insecure with moderate hunger	20 (14.8)	5 (3.7)		
		Food insecure with severe hunger	6 (4.4)	0 (0)		
Depression status	Not depressed		76 (56.3)	99 (73.3)	1	0.003* 0.004^○^
	Depressed		59 (43.7)	36 (26.7)	2.1 (1.28–3.55)	
Educational level	Diploma and higher		38 (28.1)	66 (48.9)	1	< 0.001* 0.001^○^
	Under diploma		97 (71.9)	69 (51.1)	2.4 (1.47–4.04)	
Economic status	Good (wealthy & average)		59 (43.7)	85 (63.0)	1	0.002* 0.002^○^
	Poor		76 (56.3)	50 (37.0)	2.1 (1.34–3.56)	
Family size	Less than 5 people		53 (39.3)	72 (53.3)	1	0.020* 0.021^○^
	5 people and more		82 (60.7)	63 (46.7)	1.7 (1.09–2.86)	
Number of children	Less than 4 children		50 (37.0)	73 (54.1)	1	0.005* 0.005^○^
	4 children and more		85 (63.0)	62 (45.9)	2.0 (1.23–3.25)	
Having children under 18	No		83 (61.5)	105 (77.8)	1	0.004* 0.004^○^
	Yes		52 (38.5)	30 (22.2)	2.1 (1.28–3.73)	
Income status	≥6,000,000 riyal IRN		61 (45.2)	92 (68.1)	1	< 0.001* < 0.001^○^
	< 6,000,000 riyal IRN		74 (54.8)	43 (31.9)	2.5 (1.58–4.26)	
Consumed oil type	Liquid		72 (53.3)	104 (77.0)	1	< 0.001* < 0.001^○^
	Solid and liquid		63 (46.7)	31 (23.0)	2.9 (1.73–4.96)	
BMI	18.5–24.9 (normal)		48 (35.6)	73 (54.1)	1	0.002* 0.002^○^
	≥25 (overweight/obese)		87 (64.4)	62 (45.9)	2.1 (1.30–3.47)	
Marital status	Single, deceased spouse and divorced		18 (13.3)	15 (11.1)	–	0.57*
	Married		117 (86.7)	120 (88.9)		
Employment status	Housewife		90 (66.7)	75 (55.6)	–	0.06*
	Employed		45 (33.3)	60 (44.4)		
Menopause status	female	Menopause	52 (38.5)	36 (26.7)	–	0.10*
		Non-menopause	47 (34.8)	53 (39.3)		
	Male		36 (26.7)	46 (34.1)		
Gender	Female		99 (73.3)	89 (65.9)	–	0.18*
	Male		36 (26.7)	46 (34.1)		
Age (yrs)			46.8 ± 5.4	45.9 ± 5.9	–	0.19^●^
Weight (kg)			74.4 ± 9.7	71.6 ± 9.3	–	0.016^●^
Height (cm)			165.1 ± 8.2	166.9 ± 7.7	–	0.06^●^
BMI $\left(\frac{kg}{m^2}\right)$			28.0 ± 4.0	26.1 ± 3.3	–	< 0.001^●^
The amount of activity $\left(\frac{MET. Hr}{day}\right)$			36.7 ± 3.3	40.1 ± 4.9	–	< 0.001^●^
Final energy of activities $\frac{Kcal}{Hr}$			2630.5 ± 454.2	2782.3 ± 525.5	–	0.012^●^

*Chi-square test; ^○^Logistic regression; ^**●**^Independent t-test

The prevalence of FI was 66.7% in the patients with T2D and 41.5% in controls and this difference was significant (*P* < 0.05). The chance of diabetes in the food insecure participants was 2.8 (95% CI: 1.71–4.63) times more than the food secure ones. 43.7% of the patients with T2D and 26.7% of the participants without T2D were depressed (*P* < 0.05). The odds ratio of diabetes in the depressed participants was 2.1 (95% CI: 1.28–3.55) times higher. Also, the patients with T2D had significantly lower educations, economic level, and income and higher family size and the number of children than controls (*P* < 0.05). The difference of consumed oil type between the two groups was significant, so that, 53% of the patients with T2D and 77% of the controls used liquid oil. The overweight or obesity was nearly 65% among the patients with T2D and about 46% in the participants without T2D and this difference was statistically significant (*P* < 0.05). The odds ratio for diabetes in overweight/obese subjects was 2.1 (95% CI: 1.30–3.47) times higher. In addition, the differences between weight, BMI, MET, and final energy of various activities in the participants with and without T2D were significant (*P* < 0.05, Table 3).

According to the final analysis model, FI, depression, overweight/obesity, the number of children ≥4, and having children under 18 years were important independent risk factors for T2D, even after controlling for the potential confounders. The chance of T2D in the food insecure, depressed, and overweight/obese participants was 1.9 (95% CI: 1.13–3.33), 2.0 (95% CI: 1.13–3.61), and 1.8 (95% CI: 1.07–3.08) times higher (Table 4).

**Table 4:** Odds ratio (OR) and 95% confidence intervals (CI) of variables describing the important risk factors for type 2 diabetes (T2D)

Factors		OR (95% CI)	*P*-value
Food security	Food secure	1	0.016
	Food insecure	1.9 (1.13–3.33)	
Depression	Non-depressed	1	0.018
	Depressed	2.0 (1.13–3.61)	
BMI	18.5–24.9 (normal)	1	0.025
	≥ 25 (overweight/obese)	1.8 (1.07–3.08)	
Number of children	< 4	1	0.046
	≥ 4	1.7 (1.01–2.97)	
Children under 18	No	1	0.011
	Yes	2.1 (1.19–3.88)	

## Discussion

The prevalence of mild-to-severe FI in the participants with and without T2D was 66.7 and 41.5%, respectively. 43.7% of the patients with T2D and 26.7% of the participants without T2D were depressed. These differences were statistically significant. According to the final analysis model, FI, depression, overweight/obesity, number of children ≥4, and having children under 18 years were the important independent risk factors for T2D, even after controlling for the potential confounders.

Two separate cross-sectional studies had been previously conducted on the relationship between FI and diabetes in Canada and the United States of America (USA). According to the study in the USA on adults above 20 years, the prevalence of diabetes in the food secure, low food insecure, and severe food-insecure participants was 11.7, 10, and 16.1%, respectively. The prevalence of diabetes in severe food-insecure participants was significantly higher compared with the food secure and low food insecure subjects even after controlling for the potential confounders. So, FI was considered as a risk factor for diabetes [35]. In another study on patients with diabetes in Canada, the prevalence of FI among the participants with and without diabetes was 9.3 and 6.8%, and this difference was significant [36]. A case-control study in Latin America showed the chance of T2D in very low food insecure participants was 3.3 times higher compared with others [37]. The dietary intake of cheap foods with high energy and poor nutrients in food insecure subjects may play an important role in this relationship [35]. In other words, FI can lead to a decrease in costs for foods, dietary intake reduction, and change of the consumed food type [6, 38, 39]. So, the variety of consumed food decreases and the use of high-calorie foods will increase. These high-calorie foods include refined grains and trans or saturated fats and are cheaper and lower nutritional quality [40]. Thus, these dietary patterns can increase the risk of metabolic diseases such as obesity and diabetes [41].

In line with our results, four separate studies reported the prevalence of moderate-to-severe depression in the patients with diabetes 40.6% [42], 41.9% [43], 40.6% [44], and 41% [45]. However, the prevalence of depression in the general population of Iran was reported between 4.2 to 37% [46] and in the American adults above 65 years was 46.6% [47]. According to other studies in Iran, the prevalence of depression in patients with diabetes was reported 78% [32], 53% [48], and 64% [49]. The likely reason for the difference is score borderline for depression in these studies. According to the results of two separate studies, the chance of depression in patients with diabetes was 2 times more than the participants without diabetes [50, 51]. The depressed subjects are usually different from others in dietary intakes, controlling blood sugar, and self-caring [52]. Also, brain serotonergic activity and prolactin response are lower in subjects with metabolic syndrome and insulin resistant [53]. Some studies have shown emotional reactions may increase glycogenolysis, gluconeogenesis, and insulin resistance, decrease insulin secretion and subsequently increase the risk of diabetes in the patients [54]. In addition, hypothalamus-pituitary-adrenal-neurotrophin axis, neurotransmitters, and inflammatory factors are involved in pathology and the development of depression. Similarly, disorders of the immune system can also increase insulin resistance and the risk of T2D [55, 56].

In a study in 2003, a positive and significant relationship was observed between the chance of diabetes and high BMI [43]. Another study indicated that BMI < 25 kg/m^2^ can reduce the risk of diabetes [13]. In previous studies, a strong positive correlation between obesity (BMI ≥ 35 kg/m^2^) and the risk of diabetes have been reported. The overweight and obesity were related to FI in previous studies [57, 58] and so, they may indirectly increase the risk of diabetes. In the separate studies on women of California [59, 60], rural households of Malaysia [61], American adults [62], and groups of women [63], the prevalence of overweight was significantly higher in the food insecure group compared with food secure group. The most previous studies in Iran showed a positive and significant correlation between BMI and FI [18, 21, 64]. However, in some studies, BMI was not significantly related to FI [65–67]. Usually, the food insecure subjects use high calorie and cheap dietary patterns and thus are in the risk of overweight, obesity, metabolic syndrome, insulin resistance, and subsequently diabetes. Also, high BMI and obesity can increase the risk of metabolic diseases such as T2D by affecting inflammatory factors.

Only a cross-sectional study on patients with T2D in Tehran, Iran, 2010 showed a negative relationship between family size and the number of children > 3 and consuming fruits and vegetables that is a protective factor for diabetes [68]. In addition, FI is positively and significantly related to family size, the number of children [22, 63, 69–71], and having children under 18 [17, 18, 72, 73] according to different studies. The family size, number of children, and having children under 18 may affect the household food security and subsequently dietary patterns and risk of T2D. Children under 18 are not independent financially and routinely their parents provide their needs.

According to several studies, the physical activity level was negatively correlated with the risk of T2D [13, 74, 75]. Increasing physical activity can improve metabolism, insulin resistance, obesity, and T2D [76].

Several studies have shown a significant relationship between T2D and socioeconomic factors [77] although some results were controversial [78, 79]. The lower socio-economic levels may affect FI, dietary patterns, and subsequently T2D. The menopause status and risk of diabetes were related in some studies [80]. In this study, the menopause status was similar between the two groups. According to previous studies, the relationship between gender and T2D is controversial [75, 81–87]. The distribution of gender in two groups of this study was similar. Commonly, T2D increases with age [75, 79, 81, 88]. The range of age in two groups of our study was similar.

The budget limitations in FI individuals lead to purchase cheaper and high dense calorie foods; which might contribute to obesity, and increased susceptibility to chronic illnesses including depression, T2D and other medical conditions [3]. Finally, in the interpretation of the results and the potential underlying mechanisms of the association between FI and T2D, we can point to the adverse effects of the lower socioeconomic status as more number of the children and having the children under 18 years. on the increasing FI and consequently developing the BMI, depression, stress, and dietary inadequacy. Anyone of these changes can separately increase the incidence of T2D.

The strengths were first assessing FI as a risk factor for T2D, case-control design as the controls were selected from the same population with the same characteristics, and selecting the newly diagnosed patients which prevent bias. However, the limitations were the disadvantages of the case-control study such as the fact that they generally do not allow calculation of incidence (absolute risk) and slow sampling because of selecting the newly diagnosed patients and specific clinics and the lack of cooperation of some patients. We think slow sampling does not have significant effects on the results because all participants were equally likely to have been selected.

## Conclusion

According to the final analysis model, the important independent risk factors for T2D were FI, depression, overweight/obesity, and family size, even after controlling for the potential confounders. So, it would be necessary to assess FI and associated factors and depression as the likely risk factors for metabolic diseases such as T2D. However, the causality needs to further be studied by different studies, including cohorts or other case-controls.

## Data Availability

The datasets used and/or analyzed during the current study are available from the corresponding author on a reasonable request.

## Abbreviations

BMI: Body Mass Index
ERS: Economic Research Service
FBS: Fasting Blood Sugar
FI: Food Insecurity
METs: Metabolic Equivalents
OR: Odds Ratio
T2D: Type 2 Diabetes
USA: United States of America
USDA: United States Department of Agriculture
WHO: World Health Organization

## References

[1] Azizi S, Sadrzadeh Yeganeh H, Hosseini S, Ahmadi A, Daneshi Maskooni M, Safarpour M (2013). Food insecurity and some associated socioeconomic factors among women with metabolic syndrome referred to clinics of shiraz university of medical sciences. Armaghane Danesh.

[2] Daneshi-Maskooni M, Shab-Bidar S, Badri-Fariman M, Aubi E, Mohammadi Y, Jafarnejad S, Djafarian K (2017). Questionnaire-based prevalence of food insecurity in Iran: a review article. Iran J Public Health.

[3] Dorosty AR (2016). Food insecurity and chronic diseases: the editorial. Nutr Food Sci Res.

[4] Parsavala S, Dorosty Motlagh A, Daneshi Maskooni M, Eshraghian M, Siassi F (2013). Food insecurity and some associated socioeconomic factors among high school girls of Tehran. Intl Res J Appl Basic Sci.

[5] Dorosty-Motlagh AR, Safarpour P, Daneshi-Maskooni M, Hosseini M, Ranjbar Noshari F (2018). Food insecurity and primary school girl students’ intelligence quotients: a case-control study. J Res Health Sci.

[6] Safarpour M, Dorosty Motlagh A, Hosseini SM, Ranjbar Noshari F, Safarpour M, Daneshi Maskooni M (2014). Prevalence and outcomes of food insecurity and its relationship with some socioeconomic factors. Knowledge Health.

[7] Daneshi-Maskooni M, Badri-Fariman M, Habibi N, Dorosty-Motlagh A, Yavari H, Kashani A (2017). The relationship between food insecurity and esophageal and gastric cancers: a case-control study. J Res Health Sci.

[8] Daneshi-Maskooni M, Dorosty-Motlagh AR, Izadi A, Kashani A, Azizi S, Bahreini M (2013). Is food security associated to nutritional status among hospitalized Cancer and non-Cancer patients?. Intl Res J Appl Basic Sci.

[9] Dorosty-Motlagh AR, Safarpour M, Hosseini M, Safarpour M, Safarpour H, Daneshi-Maskooni M (2013). Intelligence quotients and socioeconomic factors. Intl Res J Appl Basic Sci.

[10] Gucciardi E, Vahabi M, Norris N, Del Monte JP, Farnum C (2014). The intersection between food insecurity and diabetes: a review. Curr Nutr Rep.

[11] Raymond J, Mahan L, Escott-Stump S (2008). Krause’s food & nutrition therapy.

[12] Goodman G, Hardman J, Limbird L, Goodman GA (2006). Insulin, Oral Hypoglycaemic agents and the pharmacology of endocrine pancreas. Pharmacol Basis Ther.

[13] Hu FB, Manson JE, Stampfer MJ, Colditz G, Liu S, Solomon CG (2001). Diet, lifestyle, and the risk of type 2 diabetes mellitus in women. N Engl J Med.

[14] Salmeron J, Hu FB, Manson JE, Stampfer MJ, Colditz GA, Rimm EB (2001). Dietary fat intake and risk of type 2 diabetes in women. Am J Clin Nutr.

[15] King H, Aubert RE, Herman WH (1998). Global burden of diabetes, 1995–2025: prevalence, numerical estimates, and projections. Diabetes Care.

[16] IDF MENA Members (2019). Members. [online] Idf.org. Available at: https://idf.org/our-network/regions-members/middle-east-and-north-africa/members/35-iran.html [Accessed 2 Feb. 2019].

[17] Wilde P. Food Policy in the United States An Introduction. 2nd ed. London: Routledge, Taylor & Francis Group; 2019. https://www.routledge.com/Food-Policy-in-the-United-States-An-Introduction-2nd-Edition/Wilde/p/book/9781138204003

[18] Ramesh T, Dorosty A, Abdollahi M (2010). Prevalence of food insecurity in household of shiraz and association with some of socioeconomic and population factors. Iran J Nutr Sci Food Technol.

[19] Djazayeri A, Pourmoghim M, Omidvar N, Dorostimotlagh A (1999). Food security and nutrient intakes in a group of high school girls in Tehran. Iran J Public Health.

[20] Eshraghian M, Siassi F, Djazayeri A (2007). Obesity and food security in Yazd primary school students. Tehran Univ Med J.

[21] Mohammadpour Koldeh M, Fouladvand M, Avakhkismi M (2011). Food insecurity as risk factor for obesity in Booshehrian low-income women. J South Med Persian Gulf Biomed Res Inst.

[22] Mohammadzadeh A, Dorosty A, Eshraghian M (2010). Household food security status and associated factors among high-school students in Esfahan, Iran. Public Health Nutr.

[23] Najafi B, Shooshtarian A (2005). Targeting of subsides and elimination of food insecurity: case report of Arsanjan. J Bus Res.

[24] Payab M, Dorosty A, Eshraghian M, Siassi F, Karimi T (2012). Association of food insecurity with some of socioeconomic and nutritional factors in mothers with primary school child in Rey city. Iran J Nutr Sci Food Technol.

[25] Ostadrahimi A, Mahboub S, Totonchi H, Dastgiri S, Dadgar L (2007). Prevalence rate and range of food insecurity of two dimensions visible and nonvisible hungry in Asadabad, Tabriz. Res J Lorestan Univ Med Sci.

[26] Haghdoost A, Rezazadeh Kermani M, Sadghirad B, Baradaran H (2009). Prevalence of type 2 diabetes in the Islamic Republic of Iran: systematic review and meta-analysis.

[27] Esteghamati A, Gouya MM, Abbasi M, Delavari A, Alikhani S, Alaedini F (2008). Prevalence of diabetes and impaired fasting glucose in the adult population of Iran: National Survey of risk factors for non-communicable diseases of Iran. Diabetes Care.

[28] Kaviani H, Rahimi P (2002). Problem solving in depressive patients with suicide attempts. Iran J Psychiatry Clin Psychol.

[29] Bickel G, Nord M, Price C, Hamilton W, Cook J (2000). Guide to measuring household food security. Revised.

[30] Beck AT, Ward CH, Mendelson M, Mock J, Erbaugh J (1961). An inventory for measuring depression. Arch Gen Psychiatry.

[31] Groth-Marnat G, Wright A. Handbook of Psychological Assessment, 6th Edition. Hoboken: Wiley; 2016. https://www.wiley.com/en-us/Handbook+of+Psychological+Assessment%2C+6th+Edition-p-9781118960646

[32] Kaviani H, Mousavi A (2008). Psychometric properties of the Persian version of Beck anxiety inventory (BAI). Tehran Univ Med J TUMS Publ.

[33] Aadahl M, Kjær M, Jørgensen T (2007). Associations between overall physical activity level and cardiovascular risk factors in an adult population. Eur J Epidemiol.

[34] Momenan AA, Delshad M, Sarbazi N, REZAEI GN, Ghanbarian A, AZIZI F (2012). Reliability and validity of the modifiable activity questionnaire (MAQ) in an Iranian urban adult population.

[35] Seligman HK, Bindman AB, Vittinghoff E, Kanaya AM, Kushel MB (2007). Food insecurity is associated with diabetes mellitus: results from the National Health Examination and nutrition examination survey (NHANES) 1999–2002. J Gen Intern Med.

[36] Gucciardi E, Vogt JA, DeMelo M, Stewart DE (2009). An exploration of the relationship between household food insecurity and diabetes mellitus in Canada. Diabetes Care.

[37] Fitzgerald N, Hromi-Fiedler A, Segura-Pérez S, Pérez-Escamilla R (2011). Food insecurity is related to increased risk of type 2 diabetes among Latinas. Ethnicity & disease.

[38] Olson CM (1999). Nutrition and health outcomes associated with food insecurity and hunger. J Nutr.

[39] Tarasuk VS, Beaton GH (1999). Women’s dietary intakes in the context of household food insecurity. J Nutr.

[40] Monsivais P, Drewnowski A (2007). The rising cost of low-energy-density foods. J Acad Nutr Diet.

[41] Vozoris NT, Tarasuk VS (2003). Household food insufficiency is associated with poorer health. J Nutr.

[42] Taziki SA, Bazrafsan HR, Behnampour N, Paviz M (2001). Relationship between depressive’s symptoms and diabetes. J Gorgan Univ Med Sci.

[43] Larijani B (2003). The association between depression and diabetes among diabetic patients in Shariati hospital. Iran J Diabetes Lipid Disord.

[44] Grandinetti A, Kaholokula JKA, Kamana’opono MC, Kenui CK, Chen R, Chang HK (2000). Relationship between depressive symptoms and diabetes among native Hawaiians. Psychoneuroendocrinology.

[45] Raval A, Dhanaraj E, Bhansali A, Grover S, Tiwari P (2010). Prevalence & determinants of depression in type 2 diabetes patients in a tertiary care Centre. Indian J Med Res.

[46] Hashemi MN, Zadehbagheri G, Ghafarian SH (2003). A survey on some etiologic factors related to depression among university students in Yasuj.

[47] Pratt LA, Brody DJ (2014). Depression and obesity in the US adult household population, 2005–2010. Women.

[48] Sepehrmanesh Z (2002). The prevalence of depression and its related factors among diabetic patients. J Kashan Univ Med Sci.

[49] Mazloomy S, Mirzaei A, Mohammadi S (2008). Study of depression prevalence in the patients with type II diabetes referring to Yazd diabetes research centers in 2008.

[50] De Groot M, Anderson R, Freedland KE, Clouse RE, Lustman PJ (2001). Association of depression and diabetes complications: a meta-analysis. Psychosom Med.

[51] Egede LE, Zheng D, Simpson K (2002). Comorbid depression is associated with increased health care use and expenditures in individuals with diabetes. Diabetes Care.

[52] Ciechanowski PS, Katon WJ, Russo JE (2000). Depression and diabetes: impact of depressive symptoms on adherence, function, and costs. Arch Intern Med.

[53] Muldoon MF, Mackey RH, Korytkowski MT, Flory JD, Pollock BG, Manuck SB (2006). The metabolic syndrome is associated with reduced central serotonergic responsivity in healthy community volunteers. J Clin Endocrinol Metab.

[54] Lustman P, Griffith L, Clouse R (1997). Depression in adults with diabetes. Seminars in clinical Neuropsychiatry.

[55] Donath MY, Shoelson SE (2011). Type 2 diabetes as an inflammatory disease. Nat Rev Immunol.

[56] Stuart MJ, Baune BT (2012). Depression and type 2 diabetes: inflammatory mechanisms of a psychoneuroendocrine co-morbidity. Neurosci Biobehav Rev.

[57] Kaur J, Lamb MM, Ogden CL (2015). The association between food insecurity and obesity in children—the National Health and nutrition examination survey. J Acad Nutr Diet.

[58] Alimoradi M, Ajami M, Abdollahi M, Ahari GK (2016). A review of the relationship between obesity and food insecurity. Int J Med Rev.

[59] Adams EJ, Grummer-Strawn L, Chavez G (2003). Food insecurity is associated with increased risk of obesity in California women. J Nutr.

[60] Kaiser LL, Townsend MS, Melgar-Quiñonez HR, Fujii ML, Crawford PB (2004). Choice of instrument influences relations between food insecurity and obesity in Latino women. Am J Clin Nutr.

[61] Shariff ZM, Lin KG (2004). Indicators and nutritional outcomes of household food insecurity among a sample of rural Malaysian women. J Nutr.

[62] Hanson KL, Sobal J, Frongillo EA (2007). Gender and marital status clarify associations between food insecurity and body weight. J Nutr.

[63] Townsend MS, Peerson J, Love B, Achterberg C, Murphy SP (2001). Food insecurity is positively related to overweight in women. J Nutr.

[64] Mohammadi F, Omidvar N, Houshiar Rad A, Mehrabi Y, Abdollahi M (2008). Association of food security and body weight status of adult members of Iranian households. Iran J Nutr Sci Food Technol.

[65] Isanaka S, Mora-Plazas M, Lopez-Arana S, Baylin A, Villamor E (2007). Food insecurity is highly prevalent and predicts underweight but not overweight in adults and school children from Bogota, Colombia. J Nutr.

[66] Whitaker RC, Sarin A (2007). Change in food security status and change in weight are not associated in urban women with preschool children. J Nutr.

[67] Gulliford MC, Mahabir D, Rocke B (2003). Food insecurity, food choices, and body mass index in adults: nutrition transition in Trinidad and Tobago. Int J Epidemiol.

[68] Farvid MS, Rabiee S, Homayoni F, Rashidkhani B, Arian V (2010). Determinants of fruit and vegetable consumption in type 2 diabetics in Tehran. Iran J Endocrinol Metab.

[69] Willows ND, Veugelers P, Raine K, Kuhle S (2009). Prevalence and sociodemographic risk factors related to household food security in aboriginal peoples in Canada. Public Health Nutr.

[70] Ghazi Tabatabaei M, Omidvar N, Alihosseini J, Vedadhir A (2014). The socio-demographic characteristics associated with food insecurity among vulnerable households in a district of Tehran. IAU Int J Soc Sci.

[71] Dastgiri S, Mahboob S, Tutunchi H, Ostadrahimi A (2006). Determinants of food insecurity: a cross-sectional study in Tabriz. J Ardabil Univ Med Sci.

[72] Nord M, Hopwood H (2007). Does interview mode matter for food security measurement? Telephone versus in-person interviews in the current population survey food security supplement. Public Health Nutr.

[73] Panigassi G, Segall-Corrêa AM, Marin-León L, Pérez-Escamilla R (2008). Sampaio MdFA, Maranha LK. Food insecurity as an indicator of inequity: analysis of a population survey. Cadernos de Saude Publica.

[74] Tuomilehto J, Lindström J, Eriksson JG, Valle TT, Hämäläinen H, Ilanne-Parikka P (2001). Prevention of type 2 diabetes mellitus by changes in lifestyle among subjects with impaired glucose tolerance. N Engl J Med.

[75] Ramachandran A, Snehalatha C, Kapur A, Vijay V, Mohan V, Das A (2001). High prevalence of diabetes and impaired glucose tolerance in India: National Urban Diabetes Survey. Diabetologia.

[76] Bakhtiari AF (2004). To survey of lifestyle in boy and girl students in Tehran dormitory. Master of sciences thesis in health education Tehran Univ Med Sci.

[77] Gove WR (1973). Sex, marital status, and mortality. Am J Sociol.

[78] Mostafaei D, Batebi A, Aezam K, Estebsari F, Shojaeizadeh D (2008). Comparison of diabetes type II patients life style effective factors with that of healthy people. J Shahid Sadoughi Univ Med Sci.

[79] Azimi-Nezhad M, Ghayour-Mobarhan M, Parizadeh M, Safarian M, Esmaeili H, Parizadeh S (2008). Prevalence of type 2 diabetes mellitus in Iran and its relationship with gender, urbanization, education, marital status, and occupation. Singap Med J.

[80] Toth MJ, Sites CK, Eltabbakh GH, Poehlman ET (2000). Effect of menopausal status on insulin-stimulated glucose disposal: comparison of middle-aged premenopausal and early postmenopausal women. Diabetes Care.

[81] Cowie CC, Rust KF, Byrd-Holt DD, Eberhardt MS, Flegal KM, Engelgau MM (2006). Prevalence of diabetes and impaired fasting glucose in adults in the US population: National Health and Nutrition Examination Survey 1999–2002. Diabetes Care.

[82] Torquato MTCG, Montenegro Junior RM, Viana LAL, Souza RAHG, Lanna CMM, Lucas JCB (2003). Prevalence of diabetes mellitus and impaired glucose tolerance in the urban population aged 30-69 years in Ribeirão Preto (São Paulo), Brazil. São Paulo Med J.

[83] Chou P, Liao M-J, Kuo H-S, Hsiao K-J, Tsai S-T (1994). A population survey on the prevalence of diabetes in kin-Hu, Kinmen. Diabetes Care.

[84] Sanjari M, Hedayati M, Azizi F (2004). Prevalence of type 2 diabetes mellitus in 3-19 age group in east of Tehran in 2001. Iran J Endocrinol Metab.

[85] Harris MI, Flegal KM, Cowie CC, Eberhardt MS, Goldstein DE, Little RR (1998). Prevalence of diabetes, impaired fasting glucose, and impaired glucose tolerance in US adults: the third National Health and nutrition examination survey, 1988–1994. Diabetes Care.

[86] Gourdy P, Ruidavets J, Ferrieres J, Ducimetiere P, Amouyel P, Arveiler D (2001). Prevalence of type 2 diabetes and impaired fasting glucose in the middle-aged population of three French regions-the MONICA study 1995–97.

[87] Gupta A, Gupta R, Sarna M, Rastogi S, Gupta V, Kothari K (2003). Prevalence of diabetes, impaired fasting glucose and insulin resistance syndrome in an urban Indian population. Diabetes Res Clin Pract.

[88] Delavari A, Forouzanfar MH, Alikhani S, Sharifian A, Kelishadi R (2009). First nationwide study of the prevalence of the metabolic syndrome and optimal cutoff points of waist circumference in the Middle East: the national survey of risk factors for noncommunicable diseases of Iran. Diabetes Care.

